# Transcriptional responses of *Arabidopsis thaliana *plants to As (V) stress

**DOI:** 10.1186/1471-2229-8-87

**Published:** 2008-08-06

**Authors:** Jason M Abercrombie, Matthew D Halfhill, Priya Ranjan, Murali R Rao, Arnold M Saxton, Joshua S Yuan, C Neal Stewart

**Affiliations:** 1 Department of Plant Sciences, University of Tennessee, 2431 Joe Johnson Blvd., Knoxville, TN 37996-4561, USA; 2 Biology Department, St. Ambrose University, 518 West Locust St., Davenport, IA 52803, USA; 3 Department of Animal Science, University of Tennessee, 2505 River Dr., Knoxville, TN 37996-4561, USA

## Abstract

**Background:**

Arsenic is toxic to plants and a common environmental pollutant. There is a strong chemical similarity between arsenate [As (V)] and phosphate (Pi). Whole genome oligonucleotide microarrays were employed to investigate the transcriptional responses of *Arabidopsis thaliana *plants to As (V) stress.

**Results:**

Antioxidant-related genes (i.e. coding for superoxide dismutases and peroxidases) play prominent roles in response to arsenate. The microarray experiment revealed induction of chloroplast Cu/Zn superoxide dismutase (SOD) (at2g28190), Cu/Zn SOD (at1g08830), as well as an SOD copper chaperone (at1g12520). On the other hand, Fe SODs were strongly repressed in response to As (V) stress. Non-parametric rank product statistics were used to detect differentially expressed genes. Arsenate stress resulted in the repression of numerous genes known to be induced by phosphate starvation. These observations were confirmed with qRT-PCR and SOD activity assays.

**Conclusion:**

Microarray data suggest that As (V) induces genes involved in response to oxidative stress and represses transcription of genes induced by phosphate starvation. This study implicates As (V) as a phosphate mimic in the cell by repressing genes normally induced when available phosphate is scarce. Most importantly, these data reveal that arsenate stress affects the expression of several genes with little or unknown biological functions, thereby providing new putative gene targets for future research.

## Background

Arsenic (As) is a toxic metalloid found ubiquitously in the environment [1] and is classified as a human carcinogen [2]. Currently, the US Environmental Protection Agency declares arsenic as the highest priority hazardous substance found at contaminated sites in the United States (see Availability and requirements section for URL). Naturally high levels of arsenic in drinking water have caused major human health problems in the United States, China, Argentina, Taiwan, and most notably in Bangladesh and India where tens of millions of people have been affected [3,4]. Arsenic is highly toxic at low concentrations, therefore drinking water safety standards were lowered from 50 to 10 μg/L in the U.S. [5].

Plants typically encounter arsenic in the anionic forms of arsenate [As (V)] and arsenite [As (III)], both of which have different cytotoxic effects [6]. As (III) reacts with the sulfhydryl groups of enzymes and proteins, thereby inhibiting cellular function and resulting in death [7]. Alternatively, As (V) is an analog of the macronutrient phosphate, so it competes with phosphate for uptake in the roots, as well as in the cytoplasm where it might disrupt metabolism by replacing phosphate in ATP to form unstable ADP-As [8]. Once taken up by the roots, arsenate is reduced to a more highly toxic species, arsenite, which is subsequently detoxified via soluble thiols such as glutathione and/or phytochelatins (PCs) and transported for vacuolar sequestration [9]. PCs are low molecular weight thiolate peptides of the general structure (γ-Glu-Cys)_*n*_-Gly (*n *= 2–11) and are synthesized from glutathione by the constitutively present phytochelatin synthase [10]. Both arsenate and arsenite efficiently induce the production of PCs in plants [11], however it is believed since arsenate has no affinity for the sulfhydryl groups in PCs, As (V) is reduced in the cytoplasm, resulting in As(III)-PC complexes [6]. Glutathione and PCs have been reported to form As(III)-tris-thiolate complexes in *Brassica juncea *upon exposure to As(V) [9]. Therefore, PC synthesis causes a depletion of cellular glutathione, resulting in a decreased capacity to quench reactive oxygen species (ROS) [12].

Phytoremediation has emerged as an alternative technology for removing toxic metals from contaminated soils and groundwater. The potential for phytoremediation to be an effective means of removing arsenic from contaminated sites has been demonstrated in hyperaccumulators of the *Pteris *genus [13-15] and may be enhanced by a better understanding of plant transcriptional responses to arsenic. Many plant studies have demonstrated the direct involvement of thiol-containing molecules (glutathione, phytochelatins, etc.) in arsenic detoxification, however more robust approaches (i.e. microarrays) should help clarify how arsenic affects plant physiological processes on a global scale. The goals of our study were to test our hypothesis that many genes would be differentially expressed in response to arsenate stress and to identify genes as putative players in As (V) detoxification using Arabidopsis as a model. In this paper, we investigate the transcriptional responses to As (V) in *Arabidopsis thaliana *using oligonucleotide microarrays. Our results demonstrate that As (V) stress strongly induces Cu/Zn superoxide dismutase (SOD) activity, but represses the production of Fe SODs. Our microarray data also suggest the involvement of other antioxidant genes, various transcription factors, tonoplast proteins, and proteins associated with cell wall growth. Of particular interest, we report that As (V) stress represses numerous genes induced by Pi starvation. We discuss the physiological implications of these findings, and suggest new avenues for research of arsenic metabolism in plants.

## Results

### Root growth under As (V) stress

**Figure 1:**
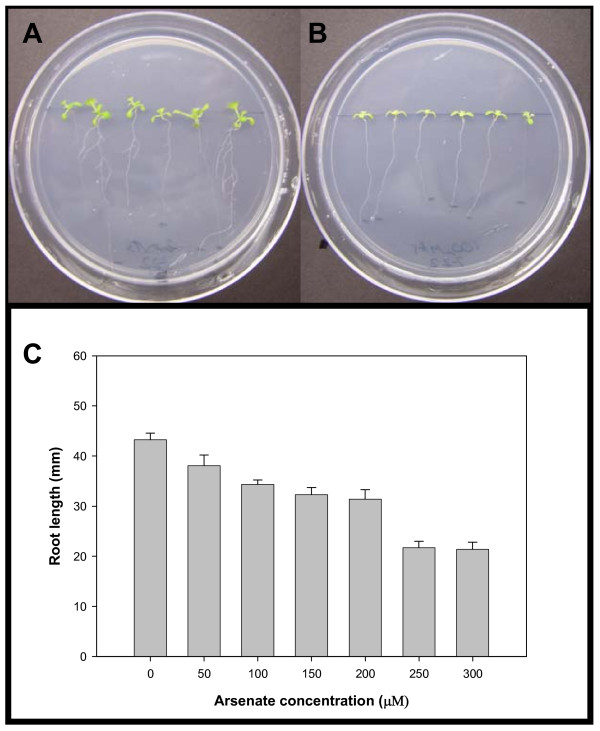
**Phenotype of arsenate stress in Arabidopsis.** Representative *Arabidopsis thaliana *"Columbia" plants grown for 10 days on MS medium containing either (Panel A) 0 μM As (V) or (Panel B) 100 μM arsenate. C. Histogram of root length vs. As (V) concentration.

Arsenate exposure resulted in reduced Arabidopsis root growth and branching (Figure 1). Exposure of 50 μM As (V) resulted in significantly reduced Arabidopsis root growth (Figure 1C). In addition to known relevant physiological data, the exposure study was used to determine suitable As (V) exposure for the microarray study. We noted no seed germination effects with regard to arsenate treatments, notably at 100 μM, the arsenate concentration used for transcriptomics experiments.

### Gene ontology for genes affected by As (V)

Forty-six genes were induced by As (V) treatment (i.e., exceeded a fold-change threshold of 1.5 and met a significance criteria of *P *< 0.001; FDR < 1%) as indicated by microarray analysis. The largest functional categories affected included unknown function, hydrolase, and antioxidant activity. Other functional categories affected by As (V) included genes with transferase, kinase, lyase, transporter, and binding activity [see Additional file 1; Table 1]. Alternatively, 113 genes were repressed by As (V) (i.e. exceeded a fold-change threshold of -1.5 and met a significance criteria of *P *< 0.001; FDR < %1), with unknown function, hydrolase, and binding activity representing the largest categories. Genes with transporter, kinase, transferase, and transcriptional regulator activity were also repressed by As (V) [see Additional file 1; Table 2]. Differentially expressed As (V)-induced and -repressed genes are listed below (Table 1 and Table 2, respectively) and complete lists of all genes affected by As (V) stress are also included as additional files [see Additional files 3 and 4]. Most interestingly, it was discovered that As (V) stress repressed transcription of many genes involved in the phosphate starvation response, and also repressed several transcriptional factors. Several genes involved in oxidative stress were also highly modulated in response to As.

**Table 1:** Gene ontology based on molecular function for induced genes of arsenic-treated *Arabidopsis thaliana *Columbia plants.

Molecular function	Gene ID	Locus	FC	RP
Antioxidant activity	Peroxiredoxin Q	at3g26060	1.53	151.4
	peroxidase	at5g64100	2.50	7.3
	peroxidase	at1g05250	1.90	49.0
	peroxidase 57 (PER57) (P57) (PRXR10)	at5g17820	1.68	56.6
	peroxidase	at1g05240	2.05	16.5
	superoxide dismutase [Cu-Zn], chloroplast	at2g28190	4.57	2.3
	superoxide dismutase [Cu-Zn], (SODCC) (CSD1)	at1g08830	2.41	8.6
	superoxide dismutase copper chaperone	at1g12520	3.16	5.2
Metal ion binding	metallothionein-like protein 1A, (MT-1A)	at1g07600	1.67	41.9
	ferredoxin, chloroplast	at1g10960	1.53	95.6
Kinase activity	leucine-rich repeat transmembrane protein kinase	at3g24240	1.59	63.2
	Cyclin-dependent protein kinase	at5g61650	1.64	66.9
Oxygen binding	non-symbiotic hemoglobin 1 (HB1) (GLB1)	at2g16060	1.59	94.2
Hydrolase activity	ATPase, BadF/BadG/BcrA/BcrD-type family	at1g30540	1.62	75.5
	myrosinase-associated protein	at1g54010	1.54	90.5
	myrosinase-associated protein	at1g54000	1.68	47.6
	xyloglucan:xyloglucosyl transferase	at4g37800	1.61	72.0
	glycosyl hydrolase family 1 protein	at3g09260	1.67	50.8
Isomerase activity	peptidyl prolyl cis-trans isomerase	at3g62030	1.64	53.1
Lyase activity	ribulose bisphosphate carboxylase small chain 2B			
	ribulose bisphosphate carboxylase small chain 3B			
Alcohol dehydrogenase activity	alcohol dehydrogenase (ADH)	at1g77120	1.74	46.5
Nitrate reductase activity	nitrate reductase 1 (NR1)	at1g77760	1.77	41.8
Sulfate reduction	5'-adenylylsulfate reductase (APR3)	at4g21990	1.53	112.1
Molecular function unknown	Photoassimilate-responsive protein	at3g54040	1.59	112.1
	Expressed protein	at1g09310	1.67	63.1
	Replication protein	At5g35260	1.62	975.3
	Hypothetical protein related to GB:AAD15331	at2g06480	1.58	81.6
	DREPP plasma membrane polypeptide-related	at5g44610	1.54	106.5
	Pentatricopeptide repeat-containing protein	at1g07590	1.62	74.6
	Meprin and TRAF domain-containing protein	at5g26280	1.60	70.8
	Expressed protein	at4g39675	1.72	86.0
	C2-domain-containing protein	at4g15740	1.76	42.7
	Expressed protein	at1g09340	1.62	78.4
	Late embryogenesis abundant 3 family protein	at4g02380	1.67	52.1
	Bet v 1 allergen family protein	at1g24020	1.54	98.4
	Universal stress protein	at3g03270	1.98	19.7
Transporter activity	Plasma membrane intrinsic protein 2B (PIP2B)	at2g37170	1.70	43.3
	Tonoplast intrinsic protein gamma	at2g36830	1.53	104.2
Glutathione transferase activity	Glutathione S-transferase GST20; Tau class	at1g78370	1.68	53.5
Cell wall structure	Glycine-rich protein	at2g05510	4.31	2.4
RNA binding	Pumilio/Puf RNA-binding domain-containing protein	At1g78160	1.60	67.7
Stress response	Drought-responsive protein (Di21)	at4g15910	1.67	69.6
Electron transport	Cytochrome B561 family protein	at5g38360	1.56	97.9
Amino acid biosynthesis	asparagine synthetase 2	at5g65010	1.74	65.1
Carbonic anhydrase activity	Carbonic anhydrase 1, chloroplast	at3g01500	1.67	51.0

RP, rank products; FC, fold change. Only genes upregulated above 1.5-fold and meeting a significance criteria of P < 0.001 and FDR of 1% are shown. Rank products analysis reveals that most significantly induced genes display the lowest RP value.

**Table 2:** Gene ontology based on molecular function for selected repressed genes of arsenic-treated *Arabidopsis thaliana *Columbia plants.

Molecular function	Gene ID	Locus	FC	RP
Catalase activity	catalase 3 (SEN2)	at1g20620	-1.59	191.7
Peroxidase activity	peroxidase	at3g49120	-1.77	165.3
	peroxidase	at5g64120	-1.84	123.2
	cationic peroxidase	at4g25980	-1.52	333.1
Oxidoreductase activity	superoxide dismutase [Fe], chloroplast	at4g25100	-5.17	1.7
	lipoxygenase	at1g72520	-2.41	242.9
	FAD-binding domain-containing protein	at1g26380	-1.50	293.0
	cytochrome p450 83B1	at4g31500	-1.71	142.3
	auxin-responsive family protein	at5g35735	-1.59	156.4
Metal ion binding	germin-like protein	at5g39160	-1.51	319.7
	germin-like protein	at5g39190	-2.13	27.3
	calcium-binding EF hand family protein	at1g76650	-2.00	50.4
	C2-domain containing protein	at4g34150	-1.53	313.4
	touch-responsive protein/calmodulin-related	at2g41100	-1.64	137.9
	ferritin 1 (FER 1)	at5g01600	-1.78	83.0
	ferritin 4	at3g56090	-1.52	201.0
	zinc finger (C2H2 type) protein	at3g46090	-1.51	191.2
	zinc finger (C2H2 type) protein	at3g46080	-1.59	178.8
	zinc finger (C3HC4 type) protein	at5g27420	-1.75	82.2
Hydrolase activity	lipase class 3 family protein	at1g02660	-1.56	218.8
	invertase/pectin methylesterase family protein	at5g62360	-1.75	85.0
	protein phosphatase 2C	at2g30020	-1.52	237.4
	phosphoric monoester hydrolase	at1g73010	-3.01	7.0
	acid phosphatase type 5 (ACP5)	at3g17790	-1.62	290.7
	phosphoric monoester hydrolase	at1g17710	-1.88	118.6
	glycosyl hydrolase family 17 protein	at3g55430	-1.53	201.2
	glycosyl hydrolase family 17 protein	at4g31140	-1.71	248.5
	glycosyl hydrolase family 17 protein	at4g19810	-1.96	99.2
	glycosyl hydrolase family 36 protein	at5g20250	-1.52	201.8
	xyloglucan endotransglucosylase/hydrolase	at4g30280	-1.63	172.2
	xyloglucan endotransglucosylase/hydrolase	at4g14130	-2.00	47.9
	xyloglucan endotransglucosylase/hydrolase	at5g57560	-1.68	97.3
	nudix hydrolase homolog 4	at1g18300	-1.54	215.9
	MERI-5 endo-xyloglucan transferase	at4g30270	-1.96	45.6
Protein binding	calmodulin-binding family protein	at4g33050	-1.78	88.6
	ankyrin repeat family protein	at5g45110	-1.58	252.0
	mitochondrial substrate carrier family protein	at4g24570	-1.50	260.2
	polygalacturonase inhibitory protein	at5g06860	-1.91	65.0
Chitin binding	hevein-like protein (HEL)	at3g04720	-1.53	499.9
Carbohydrate binding	legume lectin family protein	at3g16530	-2.03	37.8
Sugar binding	curculin-like lectin family protein	at1g78830	-1.64	138.3
ATP binding	ATP-dependent Clp protease ATP-binding subunit	at5g51070	-1.51	188.7
Jasmonic acid synthesis	allene oxide cyclase	at3g25760	-1.54	279.6
Peptidase activity	vacuolar processing enzyme gamma	at4g32940	-1.65	117.1
	subtilase family protein	at1g32970	-1.91	81.1
Ligase activity	v-box domain-containing protein	at2g35930	-1.63	205.5
	asparagine synthetase 1	at3g47340	-1.74	75.5
Transferase activity	glutathione S-transferase (GSTF6); phi class	at1g02930	-2.10	62.9
	glutathione S-transferase (GSTF7); phi class	at1g02920	-2.88	7.6
	branched-chain amino acid amino transferase 2	at1g10070	-1.60	113.8
Nutrient reservoir activity	patatin	at2g26560	-1.81	84.8
Kinase activity	serine/threonine protein kinase 19	at3g08720	-1.55	249.8
Molecular function unknown	hypothetical protein no ATG start	at3g09922	-2.15	33.6
	expressed protein	at2g25510	-1.99	96.9
	expressed protein no ATG start	at5g03545	-2.72	16.8
	expressed protein	at5g42530	-2.01	147.4
	expressed protein	at4g31570	-2.21	45.6
	expressed protein	at1g69890	-1.73	79.9
	expressed protein	at5g20790	-2.33	32.7
	expressed protein	at2g04460	-1.73	190.5
	VQ motif-containing protein	at2g22880	-1.83	57.3
	glycine-rich protein	at1g07135	-1.53	167.4
	glycine-rich protein	at3g04640	-1.81	73.9
	glycine-rich protein	at2g05540	-1.85	47.9
	glycine-rich protein	at2g05380	-1.59	146.7
	integral membrane family protein	at4g15610	-1.58	139.7
	gibberellin-responsive protein	at1g22690	-1.88	51.5
	gibberellin-regulated protein (GASA1)	at1g75750	-1.70	93.7
	dehydrin (RAB18)	at5g66400	-1.70	81.1
	unknown protein – similar to glycosyltransferase	at2g41640	-1.54	185.8
	patatin-like protein 8	at4g29800	-1.52	198.7
	phosphate-responsive protein	at5g64260	-1.52	221.0
	phosphate-responsive protein	at1g35140	-1.58	131.5
	similar to LITAF-domain containing protein	at5g13190	-1.54	293.4
Transcription factor activity	DRE-binding protein	at1g12610	-1.74	116.8
	AP2 domain-containing transcription factor	at4g34410	-2.01	50.4
	zinc finger (C2H2 type) protein	at3g46090	-1.51	191.2
	zinc finger (C2H2 type) protein	at3g46080	-1.59	178.8
	WRKY family transcription factor 33	at2g38470	-1.63	157.9
	WRKY family transcription factor 53	at4g23810	-1.55	278.3
	WRKY family transcription factor 40	at1g80840	-1.88	70.9
	NAC domain-containing protein	at5g08790	-1.53	238.7
Senescence-related	senescence-associated family protein	at5g66040	-1.55	164.0
	senescence/dehydration-associated protein	at2g17840	-1.60	256.9
	senescence-associated protein (SEN1)	at4g35770	-1.59	123.9
	SRG3 (senescence-related gene 3)	at3g02040	-2.65	11.0
Transporter activity	MATE efflux family protein	at1g61890	-1.80	108.3
Galactolipid biosynthesis	monogalactosyldiacylglycerol synthase type C	at2g11810	-1.78	130.5
Electron transport	cytochrome p450 family 94 subfamily B	at3g48520	-1.56	249.8
Guanosine tetraphosphate metabolism	RSH 2 (RELA-SPOT HOMOLOG)	at3g14050	-1.52	188.7
N-terminal protein myristoylation	band 7 family protein	at3g01290	-1.54	253.4

RP, rank products; FC, fold change. Only genes upregulated above -1.5-fold and meeting a significance criteria of P < 0.001 and FDR of 1% are shown. Rank products analysis reveals that most significantly repressed genes display the lowest RP value.

### Superoxide dismutases

SODs represented the highest ranked of both significantly induced as well as repressed genes in response to As (V) stress (Tables 1 and 2), therefore these genes presented logical primary targets for the validation of our microarray data. Results demonstrated 4.57-fold induction of a chloroplast Cu/Zn SOD (at2g28190), 2.41-fold induction of a Cu/Zn SOD (at1g08830), as well as a 3.16-fold induction of an SOD copper chaperone (at1g12520). Alternatively, Fe SOD (at4g25100) transcripts were downregulated in response to arsenic stress (-5.17-fold change). These findings were confirmed with quantitative RT-PCR (qRT-PCR) (Table 3).

**Table 3:** Comparison of microarray expression data (significance criteria of P < 0.001 and FDR of 1%) with RT-PCR data from arsenate-treated *Arabidopsis thaliana*.

Gene id	gene name	ratio^a^	ΔΔCt^b^	
			**Day 3**	**Day 10**
			
at2g28190	Cu Zn SOD (CSD2)	4.57	3.06	3.77
at1g12520	Cu Zn SOD Cu chaperone	3.16	3.14	2.44
at1g08830	Cu Zn SOD (CSD1)	2.41	4.54	2.96
at4g25100	Fe SOD (SODB)	-5.17	-2.49	-1.80
at1g73010	phosphoric monoester hydrolase	-3.01	-6.70	-2.97
at5g20790	expressed protein	-2.33	-4.85	-2.32
at2g11810	MGDG synthase type C	-1.78	-1.50	-2.44
at3g17790	acid phosphatase type 5 (ACP5)	-1.62	-4.65	-4.44
at1g17710	phosphoric monoester hydrolase	-1.88	-9.57	-3.00
at2g04460	expressed protein	-1.73	-6.04	-2.80
at3g08720	serine/threonine protein kinase 19	-1.55	-5.58	-1.82
at3g02040	senescence-related gene 3 (SRG3)	-2.65	-5.12	-1.85
at5g61650	P-type cyclin	1.64	-5.60	-3.39

^a ^Microarray data (linear fold-change)

^b ^RT-PCR data (ΔΔCt values) Arabidopsis Actin was used as a reference gene (see Materials and Methods)

*P *values for RT-PCR data were < 0.05.

**Figure 2:**
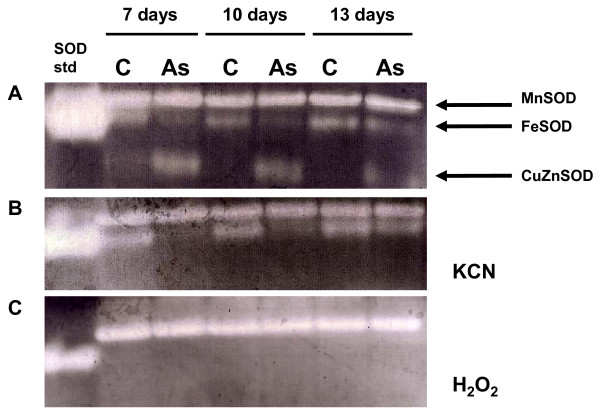
**SOD activity in *Arabidopsis thaliana *'Col' grown on medium containing 100 μM potassium arsenate.** A, superoxide dismutase activity without inhibitors, B, Gels were preincubated with KCN (which inhibits CuZn SOD), C, H_2_O_2 _added as an inhibitor (which inhibits both CuZn SOD and Fe SOD). Lane 1, purified bovine SOD positive control. Lane 2, control plants harvested at 7 days, Lane 3, arsenate-treated plants harvested at 7 days. Lane 4, control plants harvested at 10 days, Lane 5, arsenate-treated plants harvested at 10 days. Lane 6, control plants harvested at 13 days, Lane 7, arsenate-treated plants harvested at 13 days.

Based upon our observations of transcript-level changes in SOD gene expression, we performed a nondenaturing PAGE enzyme assay of superoxide dismutase activity [16] to assess whether the observed specific changes in SOD transcript correlated with enzyme activity. This method enables the distinction between the three SOD isoenzymes found in Arabidopsis (CuZnSOD, FeSOD, and MnSOD) by using inhibitors of specific SODs. Gels were preincubated with KCN, which inhibits CuZn SOD, as well as H_2_O_2_, which inhibits both CuZn SOD and Fe SOD. MnSOD is resistant to both inhibitors (Figure 2). Plants were harvested from control plates containing no arsenate and treated plates containing 100 μM arsenate at seven-, ten-, and thirteen days post-germination. Irrespective of harvest date, CuZnSOD activity was strongly induced by arsenate treatment, whereas FeSOD activity was repressed, and MnSOD showed no change in activity, therefore providing sufficient evidence to confirm our microarray results. Transcription of other antioxidant genes (i.e., peroxidases, glutathione-S transferases, catalase) were indicated by our microarray experiment as affected by arsenate stress, however these genes were not included in our qRT-PCR validation.

### Transcription factors

Our microarray experiment indicated that eight different genes encoding proteins with known transcription factor activity all displayed lower expression levels in As (V)-stressed plants (Table 2). One of these transcription factors (at1g12610) encodes a member of the DREB subfamily A-1 of the *ERF/AP2 *transcription factor family (*DDF1*). One other AP2-domain-containing transcription factor (at4g34410) that encodes a member of the ERF (ethylene response factor) subfamily B-3 of the *ERF/AP2 *transcription factor family was also repressed in response to As (V). Two zinc finger (C2H2 type) genes (at3g46090, at3g46080) encoded a ZAT7 and a protein similar to ZAT7, respectively. Also exhibiting lower expression in As (V)-treated plants were three members of the WRKY family of transcription factors (at2g38470, at4g23810, at1g80840), *WRKY33*, *WRKY53*, and *WRKY40*, respectively as well as one gene encoding NAC domain containing protein 81.

### As (V) represses genes involved in phosphate starvation response

**Figure 3:**
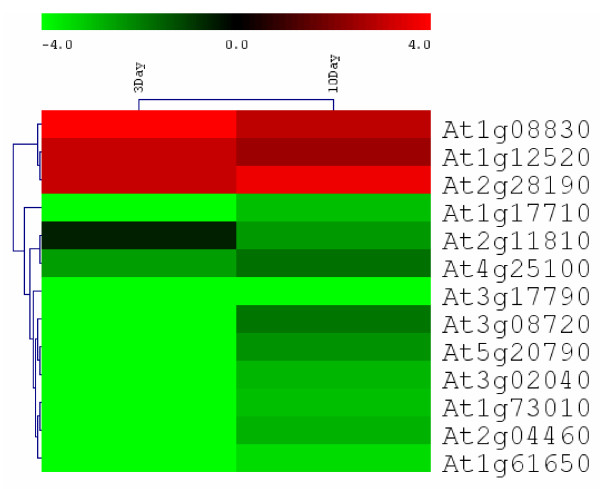
**Cluster diagram of ΔΔCt values in response to 100 μM arsenate at 3 and 10 day time points as determined by qRT-PCR.** Bright green rectangles represent strongly repressed gene expression (-4-fold and lower), whereas genes strongly induced (4-fold and higher) are shown in bright red. Arabidopsis actin was used as a reference gene to calculate ΔΔCt.

A notable transcriptional trend is that As (V) stress results in repression of some genes involved in the Pi starvation response. Of particular interest, a P-type cyclin (at5g61650) that was affected by As (V)-stress shares significant homology to the *PHO80 *gene from yeast. We performed qRT-PCR for this gene and found that its expression was actually strongly repressed at both day 3 and day 10 (Table 3; Figure 3). Three genes that were repressed by As (V) in this study have also been reported to be repressed by Pi starvation [17]. Interestingly, the three highest ranking differentially expressed genes found to be strongly induced by Pi starvation (at1g73010 > at5g20790 > at1g17710, respectively) [18], were also repressed by As (V) in our study. These genes are of particular interest on account of their unknown function. Quantitative RT-PCR confirmed that transcription of these genes was strongly repressed at both 3 day and 10 day time points. Additionally, the qRT-PCR data indicate that these genes were more repressed at day 3 than at day 10 (Table 3; Figure 3). These data also corroborated our microarray experiments, which reflect global expression ratios at 10 days post germination (Table 3).

### Sulfate assimilation

The role that thiol groups play in arsenic detoxification has been well characterized, therefore we expected to see induction of genes involved in sulfate assimilation and metabolism in response to arsenic stress. Ferredoxin (at1g10960), a key redox protein found in the chloroplast was As (V)-induced. Expression levels for another gene involved in the sulfate reduction pathway, 5'-adenylylsulfate reductase (*APR3*) (at4g21190) were also elevated in response to As (V) stress. This enzyme catalyzes the reduction of APS to sulfite using glutathione as an electron donor. Although not involved in sulfate assimilation, the cysteine-rich metal-binding protein, metallothionein (MT) 1A (at1g07600) was also induced. *Arabidopsis *knockout mutants that were generated for class 1 MTs accumulated significantly less aboveground As, Cd, and Zn, suggesting that class 1 MTs may play a role in metal and metalloid ion translocation [19].

### Genes involved in cell wall assembly, architecture, and growth

A wide range of genes encoding proteins involved in cell wall activities exhibit altered expression levels in response to As (V) (Table 1; Table 2). Peroxidases, which were indicated by microarray as affected by As (V) stress, are known to strengthen the cell wall in response to biotic stress via formation of lignin, extension cross-links, and dityrosine bonds [20]. Additionally, As (V) affected transcription of numerous xyloglucan endotransglucosylase/hydrolases (XTHs) and glycosyl hydrolase genes (Table 1; Table 2), with the majority of these exhibiting lower expression in the presence of As (V).

## Discussion

### Arsenic and oxidative stress

#### Superoxide dismutases

Increasing evidence from mammalian studies demonstrates that ROS are generated in response to exposure to inorganic forms of arsenic [21-23]. The reduction of arsenic is linked with *in vivo *and *in vitro *ROS production in mammalian cells [21], but little is known about the mechanisms by which arsenic-induced ROS generation occurs in plants. It is believed that the reduction of As (V) to As (III), which is well documented in plants, results in the production of ROS [8,24]. However, this increase in ROS may also be the result of either depletion of glutathione or inhibition of antioxidant enzymes. Plants have evolved both nonenzymatic antioxidants (i.e., glutathione, ascorbate, and carotenoids), as well as antioxidant enzymes (i.e., superoxide dismutases, catalases, and peroxidases) to manage the balance of ROS in the cell.

SODs represent a first line of defense by converting superoxide radicals to H_2_0_2_, whereas catalases and peroxidases remove H_2_O_2_. Three classes of SODs have been identified according to the active site metal cofactor: FeSOD, MnSOD, and Cu/ZnSOD. As (V) and As (III) were both shown to induce expression of glutathione S-transferases (GSTs), catalases, and SODs in *Zea mays *[24]. An increase in SOD activity was correlated with an increase in As (V) treatment in *Holcus lanatus *[12]. Higher levels of SOD, catalase, and ascorbate peroxidase were observed in *Pteris vittata*, an arsenic hyperaccumulator, than in arsenic-sensitive fern species *Pteris ensiformis *and *Nephrolepsis exaltata *[25]. These researchers concluded that arsenic-induced increases in antioxidant enzymes levels may represent a secondary defensive mechanism against oxidative stress in *Pteris vitatta *and correspond with its arsenic accumulation and lack of toxicity symptoms. It was shown that *Pteris vittata *SOD, catalase, and peroxidase levels rose sharply in response to low levels of As (V), but leveled off at As (V) levels > 20 mg kg^-1^, which was consistent with changes in biomass in the arsenic hyperaccumulator [26].

Although the strong induction of SODs in response to As (V) stress was not surprising, the dramatically lower levels of FeSODs were unexpected. We suggest the involvement of an NAC domain-containing transcription factor to explain the observed decrease in FeSOD transcription based on our microarray results (Table 2). One group recently generated transgenic plants to overexpress three different Arabidopsis NAC transcription factors and identified NAC-dependent genes using microarrays [27]. Not only was at4g25100 (FeSOD) expression found to be NAC-dependent, but transcription of other genes we have observed to be repressed by As (V) stress also appear to be dependent on NAC-domain containing transcription factors. We continue this discussion more thoroughly in the following section on transcription factors.

#### Peroxidases

Peroxidases are functionally diverse and participate in two major cycles: the hydroxylic cycle where peroxidases regulate H_2_O_2 _levels and release ROS (·OH, HOO·) and the peroxidative cycle where various substrates (e.g. phenolic compounds) are oxidized or polymerized. Their involvement in a broad range of physiological processes allows peroxidase expression in all plant organs from germination to early senescence, however they are predominantly expressed in the roots [20]. It is not surprising that peroxidases seem to be affected by arsenate stress (Table 1; Table 2), especially in consideration of the elevated SOD activity, which produces H_2_O_2 _as a product of superoxide radical dismutation.

### Transcription factors

Our microarray data corroborate those of Tran *et al*. [27], suggesting the involvement of a different NAC domain-containing transcription factor (at5g08790) in expression of FeSOD, as well as several other genes known to exhibit NAC-dependent expression. NAC proteins comprises a large gene family (> 100 members in Arabidopsis) of plant-specific transcription factors that have roles in wide-ranging processes such as development, defense, and abiotic stress response [28]. Microarray experiments were carried out on NAC-overexpression Arabidopsis mutants to discover genes exhibiting dependence on NAC transcription factors for transcription [27]. We speculate that repression of *NAC81 *(at5g08790) in As (V)-stressed Arabidopsis may be responsible for the observed repression of FeSOD (at4g25100), ferritin 1 (FER 1) (at5g01600), *XTH15 *(at4g14130), *XTH24 *(at4g30270), *erd1 *ATP-dependent Clp protease ATP-binding subunit (at5g51070), and a branched-chain amino acid amino transferase 2 (at1g10070), as these genes were reported as exhibiting NAC-dependent expression [27].

### As (V) stress represses genes induced by Pi deprivation

Although phosphate is undoubtedly one of the most biologically important nutrients, its availability in soils is quite low. Therefore, plants have evolved mechanisms to maximize Pi accessibility/availability, such as increased root hair growth, lateral root branching, and induction of phosphate transporters and phosphatases [29]. Certain phosphate starvation-induced genes have evolved to release phosphate from plasma membranes by hydrolyzing phospholipids under conditions of low Pi availability, as phospholipids comprise a major Pi pool *in planta *[30]. Conversion from phospholipids to galactolipids is one such strategy and can result from the activity of monogalactosyldiacylglycerol (MGDG) synthase or digalactosyl diacylglycerol (DGDG) synthase [31]. Arabidopsis plants expressing *MGD*2 and *MGD*3 promoter-GUS fusion constructs showed that under Pi starvation, *MGD*3::GUS was expressed in apices of serrated edges (hydathodes) and in the lateral root branch [31]. Through investigation of Arabidopsis MGDG synthase gene expression under Pi starvation, these authors showed that global changes in plant membranes under Pi deprivation are tightly regulated by Pi signaling and that signal transduction through a Pi-sensing mechanism is responsible for regulating MGDG synthase gene expression [31]. We report here that the expression of *MGD3 *(at2g11810) is lower in As (V)-treated Arabidopsis at 3 days and 10 days (Table 3; Figure 3). Therefore, it is conceivable that our observations may either reflect a Pi/As (V) sensing mechanism or simply the lower number of lateral roots in As (V)-stressed plants (Figure 1). *SENESCENCE RELATED GENE *3 (SRG3; at3g02040), a glycerophosphoryl diester phosphor-diesterase, is believed to participate in processes similar to those of the MGDG synthase genes [32]*SRG*3 had lower transcript abundance in As (V)-treated plants in our microarray study (along with other senescence-associated proteins) (Table 2), as well as, in 3 day and 10 day As (V)-treated plants (Table 3; Figure 3). A type 5 acid phosphatase (*ACP*5; at3g17790) was also repressed in our As (V)-treated plants as indicated by microarray (Table 2) and was strongly repressed in our qRT-PCR validation experiments at both 3 day and 10 day time points (Table 3; Figure 3). In Arabidopsis, *ACP*5 has been shown to be induced by H_2_O_2_, but not by paraquat or salicylic acid and is thought to be involved in both phosphate mobilization and in the metabolism of reactive oxygen species [33]. In contrast, *ACP*5 was strongly repressed by As (V) despite elevated SOD levels, which generate H_2_O_2_. Therefore, further study is required to determine the specific cause of As (V)-mediated *ACP*5 repression.

Recent investigations into the genome-scale transcriptional changes to phosphate deprivation in *Arabidopsis *have elucidated a broad range of genes involved in phosphate metabolism [17,18]. Our microarray data suggested that many genes repressed by As (V) stress have been reported by others [17,18] to be induced in response to Pi deprivation in *Arabidopsis thaliana*. Because As (V) behaves as a phosphate analog, it is likely that this observation can be explained by a saturation effect of the phosphate analog, As (V), thereby misleading metabolic and regulatory perception of the toxic metalloid as an abundant supply of Pi. However, arsenate likely disrupts critical biological processes that involve reversible phosphorylation, as well as pathways for phosphate signaling, but even under arsenate stress, Arabidopsis accumulates much higher concentrations of As in the root than is translocated to the shoot. In another study, when wild-type (Columbia ecotype) Arabidopsis plants were grown on 100 μM sodium arsenate for 3 weeks, low concentrations of arsenic were accumulated in the shoot, whereas high concentrations of arsenic were observed in roots [34]. However, when the arsenate reductase homolog (ACR2) was silenced, arsenate was translocated to the shoot at concentrations that classified as hyperaccumulation [34]. Nevertheless, the signaling mechanisms by which plants distinguish between As (V) and phosphate are unknown and other mechanisms of As detoxification and storage besides the well documented phytochelatin response [9-12] may exist.

In order to confirm the observation that As (V) stress represses genes involved in phosphate starvation/acquisition, we performed qRT-PCR on some of the more interesting candidates (Table 3; Figure 3). We are particularly interested in elucidating pathways involved in As (V) signaling in plants. The P-type cyclin (at5g61650) that was affected by As (V) (Table 3; Figure 3) shares significant homology to the *PHO80 *gene from yeast. Cyclins bind and activate cyclin-dependent kinases, which play key roles in cell division via phosphorylation of critical substrates, such as the retinoblastoma protein, transcription factors, nuclear laminar proteins, and histones [35]. Interestingly, it was demonstrated that expression of this cyclin from Arabidopsis restored the phosphate signaling pathway in a *PHO80*-deficient yeast mutant, suggesting a putative key Pi signaling role [36].

Protein kinases play crucial roles in signal transduction pathways in all eukaryotes [37]. At3g08720 (*ATPK*19) is one of two nearly identical kinase genes in Arabidopsis that encode for proteins that share high sequence homology with the mammalian 40S ribosomal protein kinases S6K1 and S6K2 [38]. *ATPK*19 was demonstrated to be the functional plant homolog of mammalian p70s6k when ectopic expression of this gene specifically phosphorylated ribosomal protein S6 derived from either plant or animal [39]. *ATPK*19 has recently been implicated as a crucial nodal point in a network evolved for integrating stress signals with plant growth regulation [40]. Lower expression levels observed for *ATPK*19 in As (V)-treated plants, which was most severe at day 3 (Table 3; Figure 3), lends us to conclude that As (V) stress may suppress plant growth through the downregulation of this growth-regulating kinase, possibly as a result of the chemical similarity between As (V) and phosphate. Alternatively, the downregulation of *ATPK*19 may result from the more general stress responses imposed by the toxic metalloid (e.g. oxidative stress, sulfhydryl group binding, etc.).

Our results are in agreement with the recently proposed ideas of Catarecha *et al*. [41] who studied an Arabidopsis mutant that displayed enhanced arsenic accumulation. These authors identified a Pi transporter (PHT1;1) mutant with a decreased rate of As (V) uptake and increased As (V) accumulation. By comparing gene expression of the mutant with wild-type plants, it was shown that in Arabidopsis, As (V) rapidly repressed genes involved in the Pi starvation response and induced the expression of other As (V)-responsive genes [41]. Interestingly, the repression of Pi starvation genes was shown to be specific for As (V), whereas the As (V)-induced genes were also induced by As (III). A model resulted that suggests arsenic acts via two separate signaling pathways [41]. Because of the chemical similarity of As (V) and Pi, As (V) fools the Pi sensor, thus initiating the repression of the Pi starvation response. Although our microarray experiments did not detect differential expression of any high-affinity Pi transporter, which may be due to differences in experimental approach, Catarecha *et al*. [41] illustrated the high sensitivity of the Pi transporter, PHT1;1, to As (V) and suggested that plants have evolved an As (V) sensing system whereby As (V) and Pi signaling pathways oppose each other to protect the plant from arsenic toxicity. Based on our results, it is conceivable that the P-type cyclin (at5g61650) and *ATPK*19 (at3g08720) may be involved in As (V) sensing, but further study is required to confirm this finding.

Our comparison of As (V)-repressed genes that have also been shown to be induced by Pi deprivation elucidate some promising candidates for future studies. For example, we are particularly interested in genes with unknown function that are strongly induced in both roots and leaves by Pi starvation (i.e. at1g73010; at1g17710; at2g04460; at5g20790; [17], supplemental data; [18]). Both at1g73010 and at1g17710 are described as phosphoric monoester hydrolases (see Availability and requirements section for URL), but to our knowledge, these have not been studied in this regard. Most recently, a study described gene networks for the Arabidopsis transcriptome based on the graphical Gaussian model of global-scale transcriptional studies [32]. In a constructed subnetwork of genes involved in phosphate starvation, SRG3, at1g73010, at3g17790 (ACP5), at2g11810, and at5g20790 were all closely linked, suggesting their critical roles in phosphate metabolism in Arabidopsis [32]. Based on their strong induction in response to Pi starvation [17,18], it is reasonable to conceive that at1g73010 and at1g17710 have evolved as Pi scavengers for increasing Pi availability. We have confirmed the downregulation of these genes in response to As (V) at both 3 and 10 day time points (Table 3; Figure 3). In this study, at2g04460 transcript levels were strongly repressed at 3 and 10 day time points, whereas at5g20790 was repressed at day 3 and day 10 (Table 3; Figure 3). Interestingly, at2g04460 encodes for a putative retroelement *pol *polyprotein that has been reported as highly expressed in salt overly sensitive (*sos*) Arabidopsis mutants [42]. Because the function of these two Pi starvation-induced genes is unknown, these putative gene candidates may provide opportunities for gaining insight into As (V)/Pi dynamics in *Arabidopsis thaliana*.

The data presented here have led to the development of new hypotheses for future research. The potential antagonistic effects of various arsenate and Pi concentrations on the expression of the aforementioned genes in Arabidopsis are poorly understood. Additionally, the efficiency of arsenate reduction and subsequent detoxification via phytochelatins or glutathione is poorly understood. Under conditions of arsenate stress (i.e., 100 μM), perhaps the cellular concentrations of arsenate surpass those that may be efficiently reduced by glutathione or arsenate reductase, thus allowing free arsenate to interfere with biological reactions that involve phosphate. Additionally, the selected arsenate concentration to employ in this study could have resulted in free arsenite after reduction *in vivo *that would likely have deleterious consequences. Therefore, it is reasonable to conceive that the observed transcriptional responses, as well as the impaired phenotype seen in this study, may be reflective of either arsenate, arsenite, or both.

## Conclusion

Our data show that in Arabidopsis, Cu/Zn SODs are strongly induced in response to As (V) stress, while Fe SOD expression is repressed. We also demonstrate that As (V) stress results in the repression of genes involved in phosphate acquisition, redistribution, and phosphorylation, which supports a recent study [41] that suggests As (V) and Pi signaling pathways act in opposition to protect plant health. Although this study identifies some interesting targets for exploring As (V) metabolism, further studies using Arabidopsis mutants with altered expression of these genes are necessary to elucidate their biological significance, as well as to clarify new pathways involved in arsenic signaling in plants.

## Methods

### Plants and growth conditions

Seeds of *Arabidopsis thaliana *ecotype Columbia plants were surface sterilized and plated on agar-solidified MS culture medium supplemented with B5 vitamins, 10% sucrose, 2% Gelrite^®^, pH 5.8. Phosphate is supplied as 1.25 mM KH_2_PO_4 _in the culture medium. Arsenic-treated plates were supplemented with 100 μM potassium arsenate (Sigma) according to a previously determined sub-lethal growth response curve. Plates were cold stratified at 4°C for 24 hrs and then placed in a growth chamber at 25°C under a 16 hr photoperiod. At each time point (3 d, 10 d), 2 g of whole plant material (shoots + roots) was harvested from each plate, frozen in liquid nitrogen, and subjected to RNA isolation using Trizol^® ^reagent (Invitrogen, Carlsbad, CA) according to manufacturer's protocol. A total of three biological replicates were assayed (3 control, 3 treated) where each pooled 2 g sample represented a single biological replicate.

### Microarray experiments and aRNA labeling

Total RNA from six biological replicates were purified using RNeasy MiniElute columns (Qiagen, Valencia, CA). A total of 1.25 μg of purified total RNA was subjected to Aminoallyl Message Amp II kit (Ambion, Austin, TX) first strand cDNA synthesis, second strand synthesis, and *in vitro *transcription for amplified RNA (aRNA) synthesis. aRNA was purified according to manufacturers protocol (Ambion, Austin, TX) and quantified using a Nanodrop spectrophotometer. Two 4 μg samples of aRNA were labeled with Cy3 and Cy5 monoreactive dyes (Amersham Pharmacia, Pittsburgh, PA) in order to conduct a dye swap technical replicate for each biological replicate. Each aRNA sample was brought to dryness in a Speedvac and dissolved in 5 μL of 0.2 M NaHCO_3 _buffer. Five microliters of Cy3 or Cy5 (in DMSO) was added to each sample and incubated for 2 hrs in the dark at RT. Labeled aRNA was purified according to kit instructions (Ambion, Austin, TX) and quantified using the Nanodrop spectrophotometer. One-hundred pmol Cy3- and Cy5-labeled aRNA targets were denatured by incubating at 65°C for 5 min and added to a hybridization mix containing 9 μl 20× SSC, 5.4 μl Liquid Block (Amersham Pharmacia, Pittsburgh, PA), and 3.6 μl 2% SDS for a 90 μl total volume.

### Hybridization and data analysis

Microarrays comprised of 70-mer oligonucleotides obtained from the University of Arizona (see Availability and requirements section for URL) were immobilized by rehydrating the slide over a 50°C waterbath for 10 s and snap drying on a 65°C heating block for 5 s for a total of four times. Slides were UV-crosslinked at 180 mJ in a UV cross-linker (Stratagene, La Jolla, CA). The slides were then washed in 1% SDS, dipped in 100% EtOH five times followed by 3 min shaking. Slides were spun dry at 1000 rpm for 2 minutes and immediately placed in a light-proof box. The 90 μl hybridization mix was pipetted onto a microarray slide underneath a lifterslip (Lifterslip, Portsmouth, NH) and placed in a hybridization chamber (Corning, Corning, NY) overnight at 55°C. After hybridization, slides were washed in 2× SSC, 0.5% SDS for 5 minutes at 55°C, 0.5× SSC for 5 minutes at room temperature, and 0.05× SSC for 5 minutes at room temperature. Slides were then spun dry at 1000 rpm in a Sorvall centrifuge and scanned with a GenePix 4000B scanner (Axon Instruments, Inc., Union City, CA). The intensity variation was removed by fitting a loess regression using SAS 9.1 (SAS, Cary, NC). Data were log-2 transformed and statistically analyzed using rank product statistics as described by [43] to identify differentially expressed genes. Bioconductor Rank Prod package was used to perform the rank product analysis [44,45]. Significantly different genes reported in this study exhibited *P *< 0.001, as designated by the rank product analysis. The false discovery rate (FDR) [46] value obtained was based on 10,000 random permutations. Since 10,000 random permutations was very computer intensive, 1000 random permutations were performed 10 different times each time starting with a different random seed number and the average FDR value calculated was used for further analysis. The genes that had FDR values less than or equal to 0.01 were considered as differentially expressed. Data for all microarray experiments were submitted to the NCBI GEO microarray database and can be viewed under the accession GSE10425.

### Microarray Data Quality Control

Global gene expression profiling comparing arsenate-treated *Arabidopsis *plants with control was carried out to better understand the mechanisms of plant response to arsenate stress and to identify genes involved in arsenic metabolism. For microarray data quality control, we examined both dye dependent effects and distribution of the ratio after normalization. We have included an additional file that illustrates the quality of microarray experiments, as well as the overall gene expression pattern [see Additional file 2]. Additional file 2a shows the normalized M vs. A plot, which was generated as a scatter plot of log intensity ratios *M = log*_2 _*(R/G) *versus average log intensities *A = log*_2_(R*G)/2, where R and G represent the fluorescence intensities in the Cy3 and Cy5 channels, respectively [47]. As shown by the figure, Loess normalization effectively removed dye dependent effects in the microarray and rendered evenly distributed ratios across all signal intensities. The histogram suggests a normal distribution of the logarithm 2-based transformed ratio [see Additional file 2b]. Overall, the microarray experiments generated high quality data without significant dye-dependent effects and skewness of ratio distribution.

### Gene ontology analysis

Gene ontology annotations were translated from microarray data using the GO annotations bioinformatics tool available at The Arabidopsis Information Resource Web site  where results were based on molecular function.

### RT-PCR amplification

Total RNA was extracted from *Arabidopsis thaliana *ecotype Columbia grown for ten days as described for the microarray experiment. Five micrograms of total RNA was reverse-transcribed with oligo(dT)_20 _primers using the Superscript III first-strand cDNA synthesis kit (Invitrogen, Carlsbad, CA). RT PCR was performed using the ABI 7000 Sequence Detection System (Applied Biosystems, Foster City, CA). PCR was performed in a 15 μl reaction volume containing Power Sybr^® ^PCR mix (Applied Biosystems, Foster City, CA) and gene-specific primers were designed with PrimerExpress software. Actin was used as the reference gene, and the primer sequences for *Arabidopsis *actin gene were AGTGGTCGTACAACCGGTATTGT (F) and GAGGAAGAGCATTCCCCTCGTA (R). After the RT PCR experiment, Ct number was extracted for both reference gene and target gene with auto baseline and manual threshold.

### Cluster Analysis

The cluster analysis was conducted with MultiExperiment viewer Version 4.0 (TIGR, Rockville, MD) with logarithm 2 transformed ratio of treated vs. control samples from real-time PCR. The complete linkage hierarchical cluster was used to cluster the genes only. The color scheme is as shown in the figure, with repressed genes shown as green and red color indicating induced genes.

### SOD activity assay

Total soluble protein was extracted from whole *Arabidopsis *plants (root + shoot) grown on plates as described above that were harvested at each respective time point. Total soluble protein was quantified by the method of Bradford [48] using BSA as a standard and 50 μg samples were loaded. Bovine SOD (Sigma) was used in each gel to serve as a positive control for SOD activity. Following electrophoretic separation on a 10% non-denaturing polyacrylamide gel, SOD activity was determined as described by Beauchamp and Fridovich (1971) and modified by Azevedo *et al*. [49]. The gels were rinsed with DDI water and incubated in the dark for 30 min at room temperature in a reaction mixture containing 50 mM potassium phosphate buffer (pH 7.8), 1 mM EDTA, 0.05 mM riboflavin, 0.1 mM nitroblue tetrazolium and 0.3% (v/v) TEMED. Following incubation, gels were rinsed with DDI water and illuminated in water until SOD bands were visible. The gels were then immersed in a 6% (v/v) acetic acid solution to stop the reaction. To confirm specificity of Cu/Zn-SOD activity, H_2_0_2 _and KCN were used as inhibitors as described by Azevedo *et al*. [49] and modified by Vitoria *et al*. [50]. Mn-SOD is resistant to both inhibitors, Fe-SOD is resistant to KCN and inhibited by H_2_0_2_, and Cu/Zn-SOD is inhibited by both inhibitors, thus allowing classification of SOD activity. Prior to SOD staining, gels containing lanes in triplicate were cut into three parts; one gel was treated as described above, the second and third parts were incubated for 20 min in 100 mM potassium phosphate buffer (pH 7.8) containing either 2 mM KCN or 5 mM H_2_O_2_, respectively. Following incubation, gels were rinsed with DDI water and then stained for SOD activity.

## Availability and requirements

US Environmental Protection Agency: 

University of Arizona: 

The Arabidopsis Information Resource:  

## Authors' contributions

Jason Abercrombie conceived of the study, its experimental design, and overall data interpretation and drafting of the manuscript. Matt Halfhill contributed to the experimental design, data interpretation, and technical assistance. Priya Ranjan, Murali Rao, and Arnold Saxton all conducted the non-parametric statistical analyses of the microarray data and reprogrammed the RankProd package to carry out 10,000 random permutations of the data and provided intellectual input for the statistical interpretation of the data. C. Neal Stewart, Jr. supervised organization of the manuscript, provided critical analyses of the data, and gave final approval of its readiness for submission. All authors read and approved the final manuscript.

## Supplementary Material

Additional file 1 Functional characterization of differentially expressed *A. thaliana *genes in response to As (V) stress. Click here for file

Additional file 2 Microarray quality control for chips used in this study. Click here for file

Additional file 3 Complete microarray dataset for genes induced by As (V). Click here for file

Additional file 4 Complete microarray dataset for genes repressed by As (V). Click here for file
