# Supplementary material for: Transcriptional responses of Arabidopsis thaliana plants to As (V) stress
Source: BMC Plant Biol. 2008 Aug 6;8:87. doi: 10.1186/1471-2229-8-87 (PMC2547109; doi:10.1186/1471-2229-8-87)
Supplement: Additional file 1 — Functional characterization of differentially expressed A. thaliana genes in response to As (V) stress. [file 1471-2229-8-87-S1.pdf]

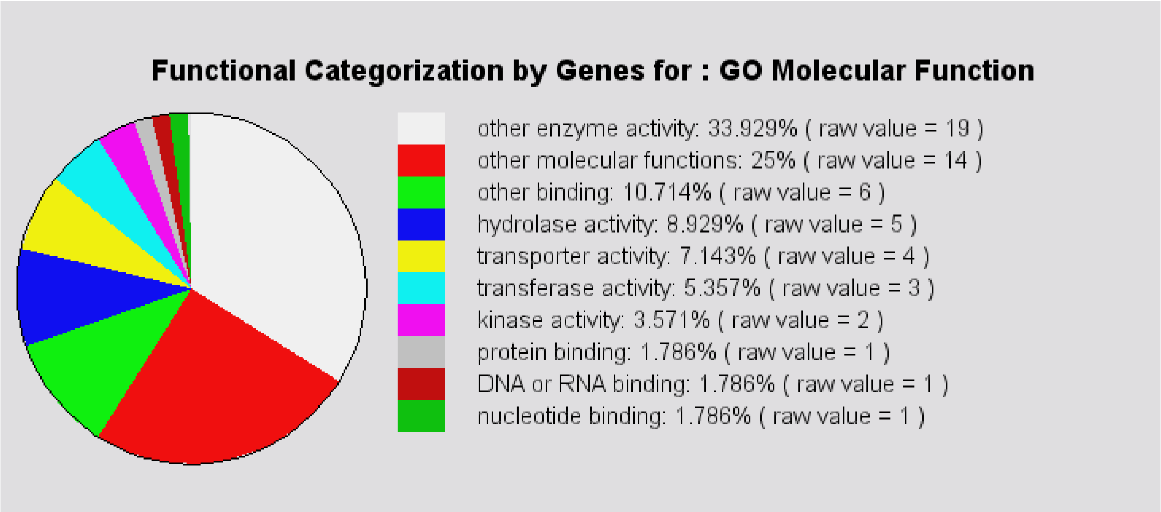

(a)

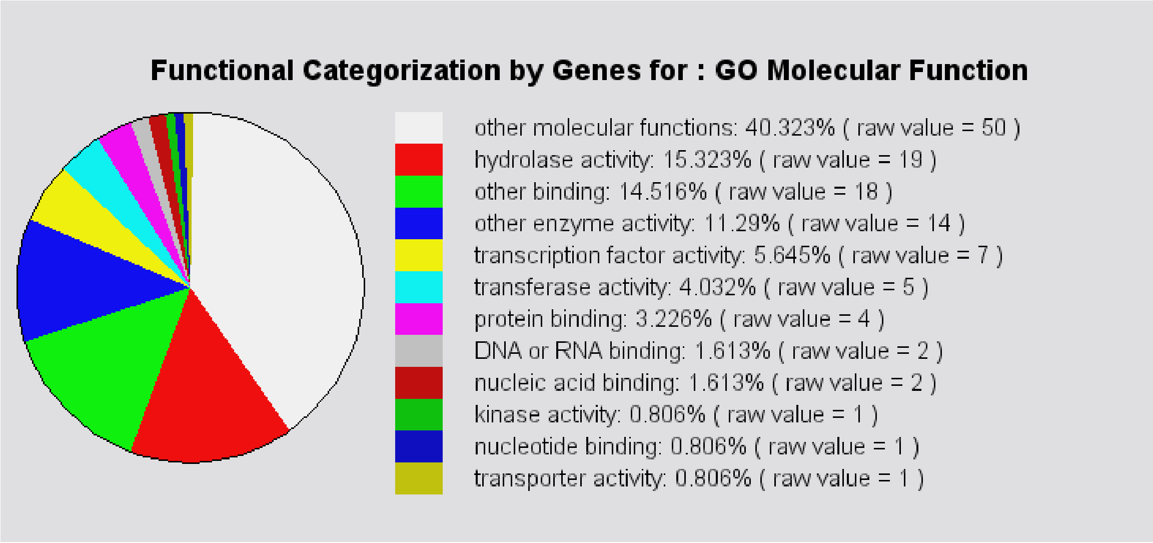

(b)

Figure S1. Functional characterization of differentially expressed *A. thaliana* genes in response to arsenate stress. A, gene ontology for genes induced above 1.5-fold. B, gene ontology for genes repressed 1.5-fold. All differentially expressed genes met a significance criteria of $P<0.001$ and had an FDR of < 1%.
