# Supplementary material for: Transcriptional responses of Arabidopsis thaliana plants to As (V) stress
Source: BMC Plant Biol. 2008 Aug 6;8:87. doi: 10.1186/1471-2229-8-87 (PMC2547109; doi:10.1186/1471-2229-8-87)
Supplement: Additional file 2 — Microarray quality control for chips used in this study. [file 1471-2229-8-87-S2.pdf]

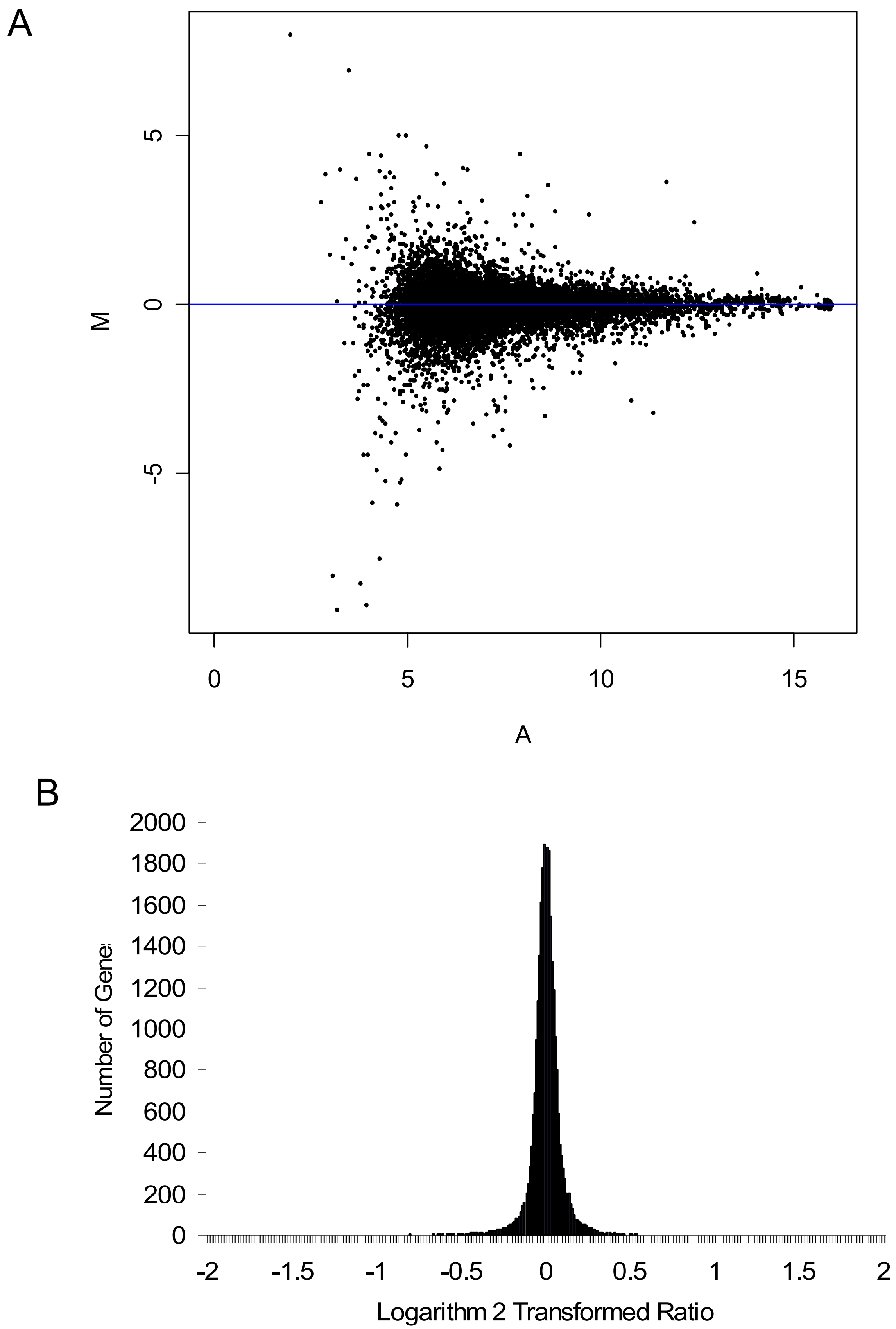

Supplementary Figure 1. Microarray data quality. A shows the M vs. A plot for normalized ratio of all six microarray slides. M vs. A plot is a scatter plot of logarithm transformed ratios *M = log2 (R/G)* plotted against average logarithm transformed intensity multiples *A = log2 (R*G)/2*, where R and G represent the fluorescence intensities in the Cy3 and Cy5 channels, respectively. B shows a histogram of distribution of logarithm 2 transformed ratios.
